# Sleep apnea predicts distinct alterations in glucose homeostasis and biomarkers in obese adults with normal and impaired glucose metabolism

**DOI:** 10.1186/1475-2840-9-83

**Published:** 2010-12-01

**Authors:** Maria Pallayova, Kimberley E Steele, Thomas H Magnuson, Michael A Schweitzer, Nathan R Hill, Shannon Bevans-Fonti, Alan R Schwartz

**Affiliations:** 1 Johns Hopkins Sleep Disorders Center, Division of Pulmonary and Critical Care Medicine, Johns Hopkins University, Baltimore, MD, USA; 2 Department of Physiology and Sleep Laboratory, PJ Safarik University School of Medicine, Kosice, Slovakia; 3 Bariatric Surgery Program, Department of Surgery, Johns Hopkins Bayview Medical Center, Baltimore, MD, USA; 4 Oxford Centre for Diabetes, Endocrinology & Metabolism, University of Oxford, Churchill Hospital, Oxford, UK

## Abstract

**Background:**

Notwithstanding previous studies supporting independent associations between obstructive sleep apnea (OSA) and prevalence of diabetes, the underlying pathogenesis of impaired glucose regulation in OSA remains unclear. We explored mechanisms linking OSA with prediabetes/diabetes and associated biomarker profiles. We hypothesized that OSA is associated with distinct alterations in glucose homeostasis and biomarker profiles in subjects with normal (NGM) and impaired glucose metabolism (IGM).

**Methods:**

Forty-five severely obese adults (36 women) without certain comorbidities/medications underwent anthropometric measurements, polysomnography, and blood tests. We measured fasting serum glucose, insulin, selected cytokines, and calculated homeostasis model assessment estimates of insulin sensitivity (HOMA-IS) and pancreatic beta-cell function (HOMA-B).

**Results:**

Both increases in apnea-hypopnea index (AHI) and the presence of prediabetes/diabetes were associated with reductions in HOMA-IS in the entire cohort even after adjustment for sex, race, age, and BMI (*P *= 0.003). In subjects with NGM (n = 30), OSA severity was associated with significantly increased HOMA-B (a trend towards decreased HOMA-IS) independent of sex and adiposity. OSA-related oxyhemoglobin desaturations correlated with TNF-α (r=-0.76; *P *= 0.001) in women with NGM and with IL-6 (rho=-0.55; *P *= 0.035) in women with IGM (n = 15) matched individually for age, adiposity, and AHI.

**Conclusions:**

OSA is independently associated with altered glucose homeostasis and increased basal beta-cell function in severely obese adults with NGM. The findings suggest that moderate to severe OSA imposes an excessive functional demand on pancreatic beta-cells, which may lead to their exhaustion and impaired secretory capacity over time. The two distinct biomarker profiles linking sleep apnea with NGM and IGM via TNF-α and IL-6 have been discerned in our study to suggest that sleep apnea and particularly nocturnal oxyhemoglobin desaturations are associated with chronic metabolic fluxes and specific cytokine stressors that reflect links between sleep apnea and glucose metabolism. The study may help illuminate potential mechanisms for glucose dysregulation in OSA, and resolve some controversy over the associations of OSA with TNF-α and IL-6 in previous studies.

## Introduction

Obstructive sleep apnea (OSA) and impaired glucose metabolism (IGM) are closely linked to an epidemic of obesity in Western society, and both are associated with a significantly increased cardiovascular risk. Epidemiologic data from the Sleep Heart Health Study [1] suggested that OSA is associated with glucose intolerance and insulin resistance independent of obesity, and may lead to type 2 diabetes mellitus. Yet the underlying mechanisms for IGM in OSA and obesity remain less well understood.

Both increases in insulin resistance and decline in beta-cell function play a role in the development of prediabetes and subsequent type 2 diabetes [2]. Despite the well-known links between OSA and glucose intolerance, the studies examining associations between OSA and glucose homeostasis have yielded mixed results. While several clinic-based studies have demonstrated an independent association between OSA and increased insulin resistance [1,3-5], two case-control studies [6,7] did not find any evidence to support the independent link between OSA and insulin resistance. Moreover, information on the impact of OSA on insulin secretion or other aspects of glucose metabolism is scarce. In a recent study [8], we suggested that hypoxic stress of OSA may be implicated in the development of insulin resistance and steatohepatitis in severe obesity. Here we extend our previous study to further elucidate the interrelationships among OSA, impaired glucose metabolism, and measures of inflammatory regulation in severely obese patients.

A major impediment to investigating metabolic consequences of OSA is inclusion of subjects with concomitant prediabetes. The prediabetes itself is associated with both insulin- and glucose resistance, decreased insulin sensitivity, and decreased beta-cell function [9]. Most previously published studies evaluating glucose homeostasis in OSA did not account for the presence of prediabetes. Furthermore, in studies prior to 2003, researchers relied upon less strict criteria for prediabetes [10]. Therefore, it is not known whether increases in insulin resistance and decreases in beta-cell function observed in OSA are related primarily to OSA or to the otherwise high prevalence of previously unrecognized concomitant prediabetes.

An increasing body of evidence suggests that OSA has also been associated with elevations of inflammatory cytokines particularly in those who are obese. However, despite controlling for adiposity, results of several studies investigating associations between OSA and adipocytokines such as tumor necrosis factor-alpha (TNF-α) and interleukin-6 (IL-6) are inconclusive. Yet none has examined associations between these biomarkers and OSA in relatively healthy adults across a range of glucose tolerances from normal to prediabetes and diabetes while controlling for the degree of obesity.

The goal of the present study was to explore the mechanisms and biomarker profiles linking OSA with glucose dysregulation in severe obesity. We hypothesized that OSA independent of obesity is associated with distinct alterations in glucose homeostasis and distinct biomarker profiles in NGM and IGM. To address this hypothesis, we recruited a group of severely obese subjects for study, since their adiposity made them prone to disturbances in glucose homeostasis and inflammatory biomarkers that could be associated with OSA.

## Subjects and Methods

### Study Design and Setting

This was a cross-sectional study conducted in 2003-2009 in the Johns Hopkins Sleep Disorders Center and the Clinical Research Unit at Johns Hopkins Bayview Medical Center. The protocols were approved by the Western Institutional Review Board. The research was conducted in accordance with the ethical standards of the institutional and national committee on human experimentation. Written informed consent was obtained from all subjects before participating in the study.

### Study Population

Participants were recruited from the Johns Hopkins Center for Bariatric Surgery in Baltimore, MD. Inclusion criteria were age >21 years and a body mass index (BMI; defined as weight in kilograms divided by the square of the height in meters) ≥35 kg/m^2^. Exclusion criteria included pregnancy, acute or chronic infectious or inflammatory disease, unstable cardiovascular disease, chronic obstructive pulmonary disease, asthma, history of thyroid disease, heart attack, stroke or surgery during the past 6 months, current systemic use of steroids, non-steroidal anti-inflammatory drugs, antibiotics, antivirals, antirheumatic drugs, hormonal contraceptives or other medications that may influence an individual's biomarker response to OSA and metabolic disturbances.

Of the 118 subjects initially recruited from the bariatric clinic, 45 were identified for inclusion. Of the entire cohort of 45 subjects, 36 (80%) were women (28 premenopausal, 8 postmenopausal) and 9 (20%) were men. Thirty subjects (66.7%) had NGM and 15 (33.3%) had IGM.

Anthropometric measurements, overnight polysomnography (PSG), and blood tests were performed in all study participants.

### Metabolic Assessment

Obesity was considered mild if the BMI was 27-30 kg/m^2^, moderate if 30-35 kg/m^2^, severe if 35-40 kg/m^2^, morbid if 40-50 kg/m^2^, super obesity if 50-60 kg/m^2^, and super, super obesity if the BMI was >60 kg/m^2 ^[11].

NGM was defined if the subject had no previous history of prediabetes or diabetes and a fasting serum glucose level <5.6 mmol/L. IGM was defined if one of the following was true: 1) a history of prediabetes or diabetes, 2) current use of an anti-diabetic medication, or 3) fasting serum glucose level ≥5.6 mmol/L.

Homeostatic model assessment (HOMA) was used to quantify estimates of steady state insulin resistance (HOMA-IR), insulin sensitivity (HOMA-IS), and pancreatic beta-cell function (HOMA-B) as percentages of normal reference populations [12]. The updated HOMA2 model [13] incorporated in a computer program HOMA Calculator v2.2.2 [14] was utilized in this study. The model consists of a number of improvements to the HOMA1 equations [12] with mathematical representations of the tissues and organs involved in glucose regulation. HOMA2 accounts for variations in hepatic and peripheral glucose resistance [15], increases in the insulin secretion curve for more hyperglycemic states, and introduces the contribution of circulating proinsulin [13]. As HOMA is based on steady state physiology, the HOMA2 model includes limits for glucose 3-25 mmol/L and insulin 20-400 pmol/L. Values outside these limits are considered non-steady state and cannot be assessed.

HOMA2 models could not be calculated for one man with NGM (missing insulin data) and for three women with IGM. One woman was taking exogenous insulin, which precluded use of the HOMA to assess beta-cell function. In two women, the insulin levels were outside limits for HOMA calculators; one insulin value 15.6 pmol/L was below the HOMA lower limit, one value 416 pmol/L was above the upper limit.

### Analytical Assay

A fasting blood sample was drawn on the morning after completion of the sleep study. Serum was stored at -80°C until assay. All samples and standards were run in duplicate and analyzed in the same batches.

Enzyme-linked immunosorbent assay (ELISA) was used for measurements of serum interleukin-1ß (IL-1ß), high sensitive IL-6, interleukin-8 (IL-8), TNF-α, soluble TNF-α receptor 1 (sTNFαR1), soluble TNF-α receptor 2 (sTNFαR2) [MSD, Gaithersburg, MD], leptin and soluble leptin receptor (R&D Systems, Inc., Minneapolis, MN), adiponectin (Millipore, Inc., St. Charles, MO), C-reactive protein (CRP) [R&D Systems, Inc., Minneapolis, MN], and insulin (Millipore Corp., Billerica, MA). Serum concentrations of ghrelin were determined by radioimmunoanalysis using commercial kits from Millipore, Inc. (St. Charles, MO).

Intra-assay coefficients of variation (CV) were 7.79% for IL-1ß, 7.79% for IL-6, 4.41% for IL-8, 7.71% for TNF-α, 1.31% for sTNFαR1, 2.55% for sTNFαR2, 8.08% for leptin, 3.1% for soluble leptin receptor, 2.11% for adiponectin, and 5.17% for ghrelin. Inter-assay CVs were 5.96% for IL-1ß, 5.96% for IL-6, 14.92% for IL-8, 11.76% for TNF-α, 12.64% for sTNFαR1, 6.45% for sTNFαR2, 3.87% for leptin, 7.7% for soluble leptin receptor, 1.65% for adiponectin, and 6.69% for ghrelin.

### Polysomnography

All subjects underwent a full-night sleep study to characterize their sleep and breathing pattern. All physiologic signals were digitally acquired by Somnologica software (Somnologica Studio, Medcare Flaga, Reykjavik, Iceland). Each recording was visually scored in 30-second epochs according to standard criteria [16]. Respiratory events and arousals were scored according to established criteria [16-19]. Apnea was defined as a drop in the peak thermal sensor excursion by ≥90% of baseline, lasting 10 seconds or longer. Hypopneas were identified if the nasal pressure signal excursions dropped by ≥30% of baseline for at least 10 seconds and there was a ≥4% desaturation from pre-event baseline.

The number of apneas and hypopneas per hour of sleep were calculated to obtain the apnea-hypopnea index (AHI). The oxygen desaturation index (ODI) was calculated for each subject as the total number of oxyhemoglobin desaturations ≥4% below the baseline level per hour of sleep.

OSA was diagnosed using the accepted criteria and standards of the American Academy of Sleep Medicine [17]. OSA was considered mild if the AHI was 5-15 events/hour, moderate if 15-30 events/hour, and severe if ≥30 events/hour [17].

### Statistical Analyses

The Shapiro-Wilk test was applied to test for a normal distribution. Continuous variables with normal distribution were compared with use of a Student's *t *test. Continuous variables with non-normal distributions were compared with use of the Wilcoxon matched-pairs signed-ranks test, the Wilcoxon rank-sum test, or the Mood's median test. The chi-square test was used to examine patterns between categorical variables.

The Pearson product-moment correlation coefficient (*r*), Spearman's rank correlation coefficient (*rho*), and multiple regression analyses were utilized to examine relationships among the variables of interest. Coefficients of partial determination (*partial R^2 ^*values) were calculated to explore relative effects of the variables on an outcome measure in the model.

To achieve approximate normality, the AHI and not normally distributed HOMA values were log-transformed prior to analysis according to the formula *log(x+1)*. The back-transformed values with their original units are reported for ease of interpretation.

Findings were considered to be statistically significant at the 5% level. All statistical calculations were performed using Stata statistical software release 11.0 (StataCorp LP, College Station, TX).

## Results

### Sample Characteristics and Metabolic Assessment

Table 1 shows characteristics of study participants and subgroups based on sex and glucose metabolism. The cohort was predominantly female, middle-aged, with both normal and impaired glucose metabolism, and had wide ranges of OSA severity and clinically severe obesity. Correlation analyses were utilized to identify variables and biomarkers associated with OSA.

**Table 1:** Clinical characteristics of study participants

	All	Men with NGM	Women with NGM	Women with IGM
	(n = 45)	(n = 9)	(n = 21)	(n = 15)
Age, years	36 (34-46)	36 (34-39)	36 (31-48)	42 (34-55)
White race, n (%)	30 (66.7)	5 (55.6)	14 (66.7)	11 (73.3)
**Anthropometrics**				
BMI, kg/m	47.3 (43.4-50.8)	49.1 (46.1- 55.2)	44.5 (40.5-48.2)	47.9 (43.4-52.3)
Severely obese, n (%)	6 (13.3)	0 (0)	5 (23.8)	1 (6.7)
Morbidly obese, n (%)	26 (57.8)	5 (55.6)	12 (57.1)	9 (60)
Super obese, n (%)	12 (26.7)	4 (44.4)	3 (14.3)	5 (33.3)
Super, super obese, n (%)	1 (2.2)	0 (0)	1 (4.8)	0 (0)
Neck, cm	41 (39.1-45.5)	49.2 (45.2-53)	39.4 (37-41.5)	41.3 (40.2-48)
Waist, cm	131.1 (122.3-144.2)	152 (143.7-161.8)	122.8 (113.6-134.3)	131.5 (125-136)
Waist-to-hip ratio	0.93 (0.87-0.98)	1.05 (0.98-1.09)	0.92 (0.86-0.96)	0.93 (0.89-0.94)
Sagittal abdominal diameter, cm	31 (28.5-35)	35 (29-36)	30 (28-33)	32 (29-35)
**Metabolic outcomes**				
Glucose, mmol/L	5.2 (4.8-5.8)	4.8 (4.7-5.2)	4.8 (4.6-5.1)	6.2 (5.8-6.8)
Insulin, pmol/L	89 (45.5-116.6)	107.6 (70.4-168.4)	75.9 (44.9-91.8)	97.3 (69.7-127)
HOMA-IR, %	1.7 (1-2.2)	2.0 (1.3-3.1)	1.4 (0.8-1.7)	2.0 (1.6-2.6)
HOMA-IS, %	60 (45.3-104.1)	49.6 (33.15-96.9)	71.8 (58.9-121.8)	50.9 (38.3-65.5)
HOMA-B, %	110.4 (90.3-159)	152.2 (112.05-210.1)	134.1 (97.8-173.4)	97.5 (80.7-109.2)
CRP, mg/L	8.1(4.8-11)	6.8 (4.5-9.2)	7.3 (3.7-10.1)	9.6 (7.6-14.3)
**Adipocytokines**				
IL-1ß, pg/mL	0.41 (0.38-0.5)	0.44 (0.4-0.56)	0.4 (0.36-0.5)	0.41 (0.38-0.48)
IL-6, pg/mL	1.95 (1.33-2.69)	1.63 (0.96-2.18)	1.67 (1.33-2.63)	2.33 (1.49-3.98)
IL-8, pg/mL	10.85 (8.36-13.15)	11.41 (10.85-13.15)	8.98 (6.9-13.24)	11.84 (9.16-12.99)
TNF-α, pg/mL	2.46 (2.03-3.16)	3.1 (2.36-3.2)	2.3 (1.93-3.45)	2.5 (2.13-2.96)
sTNFαR1, ng/mL	3.25 (2.28-4.33)	2.78 (2.02-4.30)	3.24 (2.18-4.11)	3.65 (2.79-4.41)
sTNFαR2, ng/mL	5.13 (4.22-6.72)	5.17 (4.16-6.53)	4.54 (4-6.62)	6.23 (4.55-6.78)
Leptin, μg/L	73.5 (56.7-94.9)	48.4 (43.4-61.5)	81.3 (72.3-103.0)	85.1 (47.2-95.9)
Leptin receptor, μg/L	16.2 (14.5-18.6)	14.5 (13.4-15.7)	16.4 (16.0-18.6)	16.6 (15.1-21.2)
Ghrelin, pg/mL	736.7 (589-983.2)	622.6 (533.8-720.7)	682.5 (573.6-946.7)	900.2 (690.9-1091.1)
Adiponectin, μg/mL	8.4 (6-10.6)	7.4 (3.6-9.1)	10.2 (7.7-11.6)	6.8 (5.8-11.4)
**Sleep architecture**				
Total sleep time, min	398 (365-436)	359 (338-403)	398 (365-436)	412 (373-460)
Sleep efficiency, %	87 (81.8-93.2)	89 (79.7-96.5)	86 (83-91)	90 (84.5-96)
WASO, min	37.5 (16.5-71)	43.1 (13.5-81.5)	40.5 (16.5-58.5)	29.2 (16.5-71)
N1, %	10.6 (7.7-16.2)	11.0 (8.7-24.9)	12.7 (8.1-16.3)	8.4 (5.2-13.8)
N2, %	59.7 (53.4-65.3)	55.3 (52.1-66.4)	55.7 (48.7-63.8)	64.3 (59.9-68.5)
N3, %	11.1 (2.4-16.7)	2.9 (0-11.2)	16.5 (2.4-19.7)	10.3 (5.4-13.1)
REM sleep, %	15.6 (11.2-18.9)	15.9 (14.2-19.9)	14.4 (10.4-17.7)	16.6 (12.9-22.3)
**PSG results**				
Total AHI, events/h	14.2 (6.4-22.9)	48 (22.5-71)	9 (4.2-16.5)	16.1 (4.9-22.9)
Obstructive apnea index, events/h	11.9 (5.5-18.9)	31.3 (17.7-57.6)	7.4 (4.2-11.9)	14.3 (4.9-18.3)
Central apnea index, events/h	0.24 (0-0.85)	0.41 (0-1.42)	0.25 (0-1.06)	0.23 (0-0.64)
Mixed apnea index, events/h	0.02 (0-0.39)	0.25 (0-0.75)	0 (0-0.36)	0 (0-0.33)
NREM AHI, events/h	9.6 (4.4-17.5)	44.1 (21.3-74.4)	5.2 (2.8-12)	9.6 (3-15.1)
REM AHI, events/h	32.3 (10.5-50.3)	53.4 (34.15-75.15)	23.3 (5.4-38.8)	32.8 (17.3-50.3)
▪ No OSA, n (%)	10 (22.2)	0 (0)	6 (28.5)	4 (26.7)
▪ Mild OSA, n (%)	14 (31.1)	2 (22.2)	9 (42.9)	3 (20)
▪ Moderate OSA, n (%)	13 (28.9)	2 (22.2)	5 (23.8)	6 (40)
▪ Severe OSA, n (%)	8 (17.8)	5 (55.6)	1 (4.8)	2 (13.3)
ODI, events/h	7.7 (3.8-16.6)	27.8 (10.3-69.6)	4.3 (2.2-9.4)	9.3 (4.2-18.4)
MeanSaO_2_, %	94.9 (93.5-95.9)	94.5 (89.4-95.9)	95.1 (93.7-95.9)	95.2 (93.5-95.4)
LowSaO_2_, %	91.4 (89.8-92.7)	90.1 (83-91.9)	92.5 (90.1-93.2)	91.6 (88.0-92.6)
MinSaO_2_, %	85 (82-88)	82 (68-85)	87 (83-88)	84 (72-88)
ΔSaO_2_, %	4.5 (4-5)	4.9 (4.8-11.3)	4.2 (3.6-4.6)	4.6 (4.0-6.6)

*Data are expressed as medians (IQR) if not otherwise stated. *NGM*, normal glucose metabolism. *IGM*, impaired glucose metabolism. *WASO*, wake after sleep onset. *N1, N2, N3*, stages of NREM sleep.

Table 2 displays results of the bivariate analyses in all study participants and in subgroups based on sex and glucose metabolism. The OSA severity was associated with male sex, central adiposity, and with alterations in glucose homeostasis. Importantly, the correlations of AHI with insulin and HOMA estimates observed in the entire cohort were driven by significant correlations found only in subjects with NGM.

**Table 2:** Correlations of OSA severity with clinical characteristics

	All (n = 45)	Women (n = 36)	Men^+ ^(n = 9)	All NGM (n = 30)	Women NGM (n = 21)	Women IGM (n = 15)
**AHI with:**						
**male sex**	0.49**	--	--	0.59**	--	--
**BMI**	0.29	0.29	-0.42	0.33	0.32	0.16
**neck circumference**	0.56**	0.53**	-0.02	0.57**	0.57*	0.47
**waist circumference**	0.46**	0.35*	-0.15	0.54**	0.41	0.19
**sagittal abd. diameter**	0.32*	0.42*	-0.39	0.48*	0.70**	-0.04
**insulin**	0.51**	0.49**	0.71*	0.63**	0.50*	0.48
**HOMA-IR**	0.47**	0.44*	0.72*	0.54**	0.48*	0.35
**HOMA-B**	0.43*	0.35*	0.71*	0.63**	0.64**	-0.10
**HOMA-IS**	-0.47**	-0.45*	-0.67	-0.55**	-0.48*	0.36
**leptin**	-0.39*	-0.24	-0.15	-0.46*	-0.22	-0.23
**adiponectin**	-0.25	-0.16	-0.15	-0.46*	-0.36	0.15
***LowSaO_2 _with:***						
**TNF-α**	-0.29	-0.35*	-0.22	-0.31	-0.44*	-0.13
**sTNFαR2**	-0.23	-0.34*	-0.001	-0.18	-0.47*	-0.28
**IL-6**	-0.20	-0.18	-0.66	-0.01	0.19	-0.55*

The Pearson product-moment correlation coefficients and Spearman's rank correlation coefficients. *NGM*, normal glucose metabolism; *IGM*, impaired glucose metabolism; *AHI*, apnea-hypopnea index; *LowSaO_2_*, mean low oxyhemoglobin saturation during sleep.

*P < 0.05; **P < 0.005

^+^Men with NGM only

Multivariable regression analyses were conducted to determine whether unadjusted associations persisted after controlling for potential confounders. In a model adjusting for sex, race, age, and BMI, both increases in AHI and the presence of prediabetes/diabetes were associated with reductions in HOMA-IS (*P *= 0.003) in the entire cohort. The AHI explained 20.4% (*P *= 0.006) and the IGM explained 13.8% (*P *= 0.026) of the variance in HOMA-IS in this model. Similar linear associations with HOMA-IS were observed when AHI was replaced by ODI (*P *= 0.011), degree of oxyhemoglobin desaturation associated with each disordered breathing event (ΔSaO_2_) (*P *= 0.012), minimum oxyhemoglobin saturation during sleep (MinSaO_2_) (*P *= 0.016) or variability of oxyhemoglobin saturation during sleep as measured by SD and CV (both *P *= 0.015).

**Figure 1:**
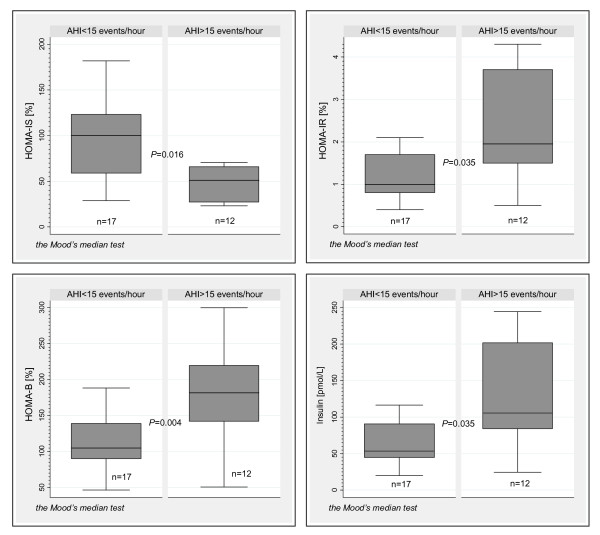
**HOMA estimates and insulin levels in subjects with normal glucose metabolism stratified by OSA severity**. The OSA severity stratification was based on AHI 15 events/hour. Box plots displays boxes bordered at the 25^th ^and 75^th ^percentiles of the *y *variable with *a median line *at the 50^th ^percentile. Whiskers extend from the box to the upper and lower *adjacent values *and are capped with *an adjacent line*.

**Figure 2:**
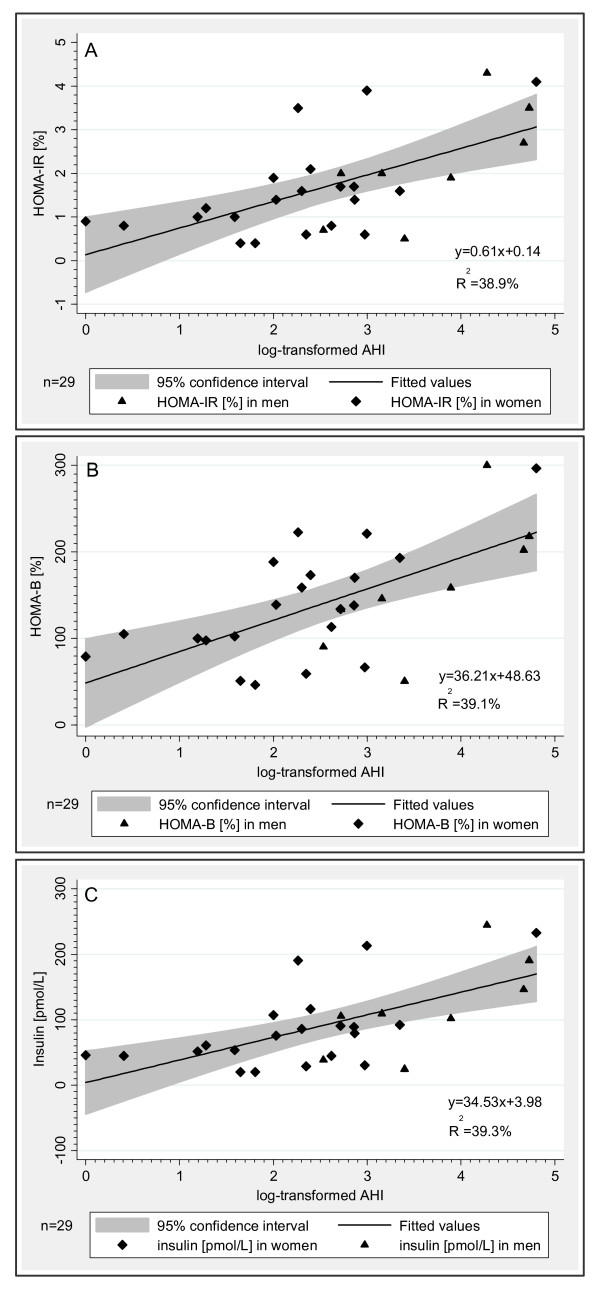
**Associations between OSA severity and insulin homeostasis in subjects with normal glucose metabolism**. Figures 2A-C illustrate predictions of HOMA-IR, HOMA-B, and insulin levels calculated from log-transformed AHI using a linear regression in women *(solid diamond) *and men *(solid triangle) *with normal glucose metabolism. The thick line represents *the line of best fit *for a set of data points. The shaded gray area represents *the 95% confidence interval *around the best-fit line.

To further explore the alterations in glucose homeostasis associated with OSA in NGM, we compared HOMA estimates and insulin levels in the subjects with NGM stratified by OSA severity. Utilizing an AHI cut-off of 15 events/hour, we found significant elevations in median HOMA-IR, HOMA-B, and insulin levels and significant reductions in median HOMA-IS in subjects with AHI > 15 events/hour compared to those with AHI < 15 events/hour *(*Figure 1) despite no significant differences in BMI (*P *= 0.213) and age (*P *= 0.450). Nevertheless, the individuals with AHI > 15 events/hour had larger neck circumference (*P *= 0.030) and sagittal abdominal diameter (*P *= 0.027), and were more likely to be male (*P *= 0.013). No significant differences were observed for CRP or adipokine levels between the two NGM subgroups. Bivariate and linear regression analyses *(*Table 2 and Figure 2) confirmed that increases in HOMA-B and insulin levels were strongly associated with OSA severity in both men and women with NGM and for the NGM group as a whole.

Even after adjusting for sex and BMI, the increases in AHI in subjects with NGM were associated with increases in HOMA-B (*P *< 0.001), HOMA-IR (*P *< 0.001), and insulin levels (*P *< 0.001). Results were similar when BMI was replaced by waist circumference in the regression models. Other measures of OSA severity, including ODI, MinSaO_2_, ΔSaO_2_, and SD and CV of oxyhemoglobin saturation during sleep were significantly linearly associated with increased HOMA-B in subjects with NGM independent of sex and BMI. No significant associations were found between HOMA estimates and mean oxyhemoglobin saturation during sleep.

### Matched-pair Case-control Analysis of Biomarker Profiles

Employing a matched-pair design, we further explored biomarker profiles associated with OSA in subjects with NGM and IGM. We have identified 15 female-female pairs matched individually for age (*P *= 0.231), BMI (*P *= 0.215), waist circumference (*P *= 0.149), and AHI (*P *= 0.191). The individually matched pairs were distinct on the basis of glucose metabolism status and fasting serum glucose (*P *< 0.001). The case group consisted of 12 women (80%) with prediabetes and 3 (20%) with type 2 diabetes. The control group consisted exclusively of women with NGM.

In a univariate analysis, we confirmed that subjects with IGM had reductions in both HOMA-B (*P *= 0.036) and HOMA-IS (*P *= 0.041) compared to those with NGM. There were trends towards increased insulin levels (*P *= 0.069) and increased HOMA-IR (*P *= 0.050) in the case group. No significant differences were observed between the case and control groups for the absolute cytokine levels.

**Figure 3:**
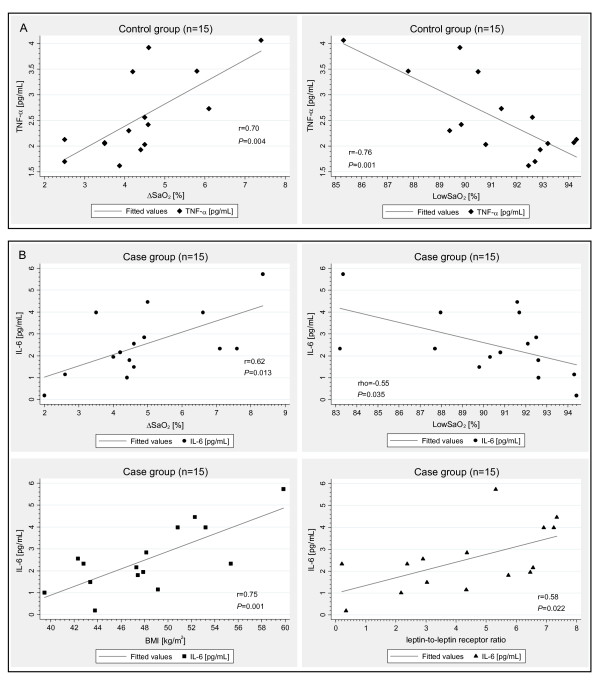
**Biomarker profiles associated with OSA in normal vs. impaired glucose metabolism**. Figure 3A presents the correlations of LowSaO_2 _and ΔSaO_2 _with TNF-α level *(solid diamond) *in the control group of 15 women with normal glucose metabolism. Figure 3B displays the correlations of LowSaO_2 _and ΔSaO_2 _with IL-6 level *(solid circle) *and the correlations of IL-6 with BMI *(solid square) *and leptin-to-leptin receptor ratio *(solid triangle) *in the case group of 15 women with impaired glucose metabolism. The fitted curve represents *a linear regression fit *between the two variables. *r *= Pearson product-moment correlation coefficient; *rho *= Spearman's rank correlation coefficient.

Through bivariate association analyses, we identified two distinct biomarker profiles associated with OSA in subjects with NGM (Figure 3A) and IGM (Figure 3B). In the control group with NGM, the mean low oxyhemoglobin saturation during sleep (LowSaO_2_) and ΔSaO_2 _were strongly correlated with TNF-α and TNF-α was strongly correlated with sTNFαR2 (rho = 0.73; *P *= 0.002), whereas in the case group with IGM, there was a strong correlation of LowSaO_2 _and ΔSaO_2 _with IL-6. Furthermore, the IL-6 in the case group was strongly positively associated with BMI and leptin-to-leptin receptor ratio.

In a multivariate linear regression analysis in the control group, the LowSaO_2 _was associated with TNF-α even after adjustment for BMI and age (*P *= 0.011). When BMI was replaced by waist circumference in the regression analysis, the LowSaO_2 _maintained an independent association with TNF-α (*P *= 0.013).

In the case group, the IL-6 was positively linearly associated with leptin-to-leptin receptor ratio, BMI, and LowSaO_2 _or AHI (both *P *< 0.001). Positive associations of IL-6 with leptin-to-leptin receptor ratio, BMI, and AHI persisted after adjustment for age (*P *< 0.001). In this model, the leptin-to-leptin receptor ratio explained the highest proportion of the variance of IL-6 (52.8%; *P *= 0.007). The BMI explained 48.4% (*P *= 0.012) and the AHI 44.1% (*P *= 0.019) of the variance of IL-6 in the case group when all other variables were included in the model. Results were similar when BMI was replaced by waist circumference in the regression model (*P *< 0.001).

## Discussion

There were three major findings in the present study. First, as expected OSA severity was associated with alterations in glucose homeostasis in the entire cohort of severely obese men and women across a range of glucose tolerances. The observed associations of OSA severity with alterations in glucose homeostasis were, however, driven by significant correlations found only in the subjects with NGM. Second, a stratified analysis based on glucose metabolism confirmed the independent linear association between OSA severity and increased basal pancreatic beta-cell function in subjects with NGM but not in those with prediabetes and diabetes. Third, we identified two distinct biomarker profiles associated with OSA in subjects with NGM and IGM that may reflect pathophysiology of glucose dysregulation in OSA and severe obesity.

Our study may help resolve inconsistencies in the current literature regarding the relationship between OSA and glucose homeostasis [1,3-7]. Several lines of evidence have emerged from our study to suggest that accounting for presence of prediabetes in studies investigating the alterations in glucose homeostasis in patients with OSA is highly relevant to achieving a better understanding of the underlying pathophysiology linking the two disorders. First, the independent associations of OSA severity with increased beta-cell function in NGM were found in our study and highlight the importance of a compensatory increase in insulin secretion as a mechanism through which beta-cell adaptation to increased insulin resistance occurs [20]. Second, our study demonstrated that subjects with IGM had both reduced beta-cell function and decreased insulin sensitivity, which confirms previous reports of a decline in pancreatic beta*-*cell function in prediabetes [2,9,20]. This finding also suggests that reduced pancreatic beta-cell function in patients with moderate to severe OSA reported in a previous study [21] may have been due to the presence of coexistent prediabetes particularly in the patients with moderate to severe OSA. Third, there were no linear associations between OSA severity and HOMA estimates in the subjects with IGM compared to individually matched controls with NGM. These findings strongly suggest that prediabetes and diabetes rather than OSA are directly associated with reduced beta-cell function in patients with IGM and severe obesity.

Our findings further suggest that pancreatic beta-cell response to OSA differs in subjects with NGM and IGM. Although both groups of severely obese individuals in our study were considerably hyperinsulinemic and insulin resistant, consistent with previous findings relating abdominal fat distribution to insulin resistance [22], OSA was directly associated with increased insulin resistance only in the subjects with NGM. It should be recognized that increased basal insulin levels are not an accurate reflection of beta-cell insulin secretion. In type 2 diabetes, basal proinsulin immunoreactivity contributes approximately 2-3 times more to insulin immunoreactivity than in healthy subjects [23]. Thus, the observed hyperinsulinemia in IGM might reflect incomplete processing of proinsulin to insulin with a combination of high proinsulin levels and low or normal true insulin levels. Although it is still not known whether nocturnal hypoxemia and increased beta-cell secretory demand are associated with a progressive increase in proinsulin/immunoreactive insulin ratio over time, data from our study suggest that OSA contributes to insulin resistance that imposes an excessive functional demand on pancreatic beta-cells, which may lead to their exhaustion and impaired secretory capacity over time.

Biomarker profiles associated with OSA via TNF-α and IL-6 have been discerned in our study, which can illuminate potential mechanisms for glucose dysregulation in OSA. TNF-α and IL-6 are critical elements in inflammatory responses. It has been shown previously that TNF-α stimulates secretion of IL-6 via an NF-kappaB-dependent pathway [24]. The opposite regulation of TNF-α and IL-6 has also been demonstrated in a recent study in rats [25] where paradoxical sleep deprivation caused an increase in serum corticosterone levels, which was associated with up-regulation of IL-6 in retroperitoneal adipose tissue and downregulation of TNF-α in mesenteric adipose tissue. TNF-α and IL-6 levels have been reported by others to be increased in patients with OSA [3,26-28] and in those with left ventricular diastolic dysfunction and glucose metabolism disorders [29]. However, no differences were found in other studies investigating associations of OSA with the cytokines [30-32]. Substantial experimental evidence and cross-sectional data suggest that IL-6 is associated with hyperglycemia, insulin resistance, and overt type 2 diabetes [33,34]. Furthermore, elevated IL-6 levels predicted the development of type 2 diabetes in a prospective case-control study [35], supporting a possible role for inflammation in diabetogenesis. Yet to date, there is no prospective study that has explored a possible role of an OSA triggered NF-kappaB-mediated TNF-α-induced IL-6 gene expression in determining glucose tolerance status in obese persons with OSA over time. Our findings suggest that OSA is closely associated with IL-6 production in IGM, whereas in the absence of IGM, it is associated with TNF-α. Importantly, our results further showed additional close associations of IL-6 with BMI and leptin-to-leptin receptor ratio in IGM. This finding supports the major role that IL-6 plays in obesity and extends it to pathophysiological processes linking obesity to IGM via leptin resistance and OSA. Finally, the finding of the two biomarker profiles associated with OSA in NGM vs. IGM may help resolve some controversy over the associations of OSA with TNF-α and IL-6 in previous studies.

There are several limitations to our study that should be considered in relation to the findings. First, its cross-sectional design precludes inferences regarding causality. Second, strict inclusion criteria and a substantially lower number of male subjects in bariatric clinic cohorts hindered enrollment of men with IGM in this study. Yet despite the small sample size of men with NGM, the observed associations between OSA severity and HOMA estimates were similar to those observed in women. Additionally, our results are consistent with the previous reports of sexual dimorphism in leptin levels [36,37]. Third, although the use of the updated HOMA2 model has been validated against a number of independent measures of insulin sensitivity and beta-cell function [38-40], as with all models, the data need to be interpreted with caution. Also, it should be recognized that HOMA is a measure of basal insulin sensitivity and basal beta-cell function and, in contrast to insulin-glucose clamps, is not intended to give information about the stimulated state. Finally, in our study, we did not perform oral glucose tolerance tests in all subjects. Therefore, the exact classification of glucose tolerance is impossible.

## Conclusions

Despite the limitations, our study demonstrated that OSA is independently associated with altered glucose homeostasis and increased basal pancreatic beta-cell function in severely obese adults with NGM. The findings suggest that moderate to severe OSA imposes an excessive functional demand on pancreatic beta-cells, which may lead to their exhaustion and impaired secretory capacity over time. The two distinct biomarker profiles discerned in our study suggest that OSA and particularly nocturnal oxyhemoglobin desaturations are associated with chronic metabolic fluxes and cytokine stressors that reflect links between OSA and glucose metabolism. The study may help illuminate potential mechanisms for glucose dysregulation in OSA, and resolve some controversy over the associations of OSA with TNF-α and IL-6 in previous studies. Further prospective studies are needed to explore the impact of OSA on pancreatic beta-cells, and to further elucidate the dynamic interactions between the adipocytokines linking sleep apnea with glucose metabolism.

## Abbreviation list

**AHI: **apnea-hypopnea index; **BMI**: body mass index; **CRP: **C-reactive protein; **CV: **coefficient of variation; **ELISA: **enzyme-linked immunosorbent assay; **HOMA: **homeostasis model assessment; **HOMA-B: **HOMA estimate of steady state beta-cell function; **HOMA-IR: **HOMA estimate of steady state insulin resistance; **HOMA-IS:**HOMA estimate of steady state insulin sensitivity; **IGM: **impaired glucose metabolism; **IQR: **interquartile range; **IL-1ß: **interleukin-1ß; **IL-6: **interleukin-6; **IL-8: **interleukin-8; **LowSaO_2_: **mean low oxyhemoglobin saturation during sleep; **MeanSaO_2_: **mean oxyhemoglobin saturation during sleep; **MinSaO_2_: **minimum oxyhemoglobin saturation during sleep; **NGM: **normal glucose metabolism; **NREM: **non-rapid eye movement; **ODI: **number of oxyhemoglobin desaturation events ≥ 4% per hour of sleep; **OSA: **obstructive sleep apnea; **PSG: **polysomnography; **r: **Pearson product-moment correlation coefficient; **REM: **rapid eye movement; **rho: **Spearman's rank correlation coefficient; **TNF-α: **tumor necrosis factor-alpha; **sTNFαR1: **soluble tumor necrosis factor-alpha receptor 1; **sTNFαR2: **soluble tumor necrosis factor-alpha receptor 2; **WASO: **wake after sleep onset; **ΔSaO_2_: ***degree of oxyhemoglobin desaturation associated with each disordered breathing *Event.

## Competing interests

**MP **received grant support (November 10, 2008 - November 9, 2009) from the European Respiratory Society, and was supported through a NIH grant (November 10, 2009 - June 30, 2010**). KES, THM, MAS, NRH**, and **SBF **declare that they have no competing interest. **ARS **received grant support (July 1, 2005 - present) from the NIH. **ARS **is a scientific advisor for Apnex Medical, Cardiac Concepts and Sova Pharmaceutical, and has stock of less than 1000 USD in Sova Pharmaceutical.

## Authors' contributions

MP participated in the design, coordination of the study, and interpretation of data, and performed the statistical analysis, and drafted the manuscript. KES participated in acquisition of data and conduct of the study. THM participated in acquisition of data and conduct of the study.MAS participated in acquisition of data and conduct of the study.

NRH participated in analysis and interpretation of data, and helped to draft the manuscript. SBF participated in the immunoassays and conduct of the study.ARS conceived of the study and participated in the design, coordination, and conduct of the study, and helped to draft the manuscript. All authors revised the article critically for important intellectual content and approved the final version.
